# Evaluation of the efficacy of Si-based agent in Rheumatoid Arthritis with complicating vestibular impairment

**DOI:** 10.1371/journal.pone.0361954

**Published:** 2026-11-06

**Authors:** Shotaro Harada, Yoshihisa Koyama, Yuki Kobayashi, Takao Imai, Yoshichika Yoshioka, Hikaru Kobayashi, Hidenori Inohara, Shoichi Shimada

**Affiliations:** 1 Department of Otorhinolaryngology-Head and Neck Surgery, Graduate School of Medicine, The University of Osaka, Suita, Japan; 2 Department of Neuroscience and Cell Biology, Graduate School of Medicine, The University of Osaka, Suita, Japan; 3 Addiction Research Unit, Osaka Psychiatric Research Center, Osaka Psychiatric Medical Center, Osaka, Japan; 4 SANKEN, The University of Osaka, Suita, Japan; 5 Graduate School of Frontier Biosciences, Osaka University, Osaka, Japan; 6 Institute for Biomedical Sciences, Iwate Medical University, Morioka, Japan; Teikyo University Hospital Mizonokuchi, JAPAN

## Abstract

**Background:**

Rheumatoid arthritis (RA) is a chronic autoimmune disease characterized by persistent joint inflammation and progressive disability. Although current therapies have improved clinical outcomes, safer and more effective therapeutic approaches remain needed. Silicon (Si)-based agents have antioxidant and anti-inflammatory properties and have shown beneficial effects in several inflammatory disease models.

**Methods:**

We evaluated the effects of oral administration of a Si-based agent in a type II collagen-induced arthritis mouse model. Arthritis severity was assessed using an arthritis index, hindlimb oedema, and the hindlimb extension reflex test. Serum type II collagen-specific autoantibody levels were also measured. Vestibular function was evaluated using the vestibulo-ocular reflex (VOR), and inner-ear structural changes were assessed by magnetic resonance imaging and histological analysis.

**Results:**

The Si-based agent reduced arthritis-related outcomes, including the arthritis index, hindlimb oedema, and joint movement restriction, and reduced serum type II collagen-specific autoantibody levels. In contrast, no significant differences between the Si-based agent and control groups were detected in VOR-related outcomes, endolymphatic space measurements, or inner-ear histological findings.

**Conclusions:**

These findings suggest that the Si-based agent may have beneficial effects on arthritis-related manifestations. However, its effects on the vestibular function-related outcomes examined in this study were not evident. Further studies are needed to clarify the relationship between RA and vestibular dysfunction and to determine the potential therapeutic effects of Si-based agent on these complications.

## Introduction

Rheumatoid arthritis (RA) is a chronic systemic autoimmune disease characterized by persistent synovial inflammation, leading to progressive joint destruction, pain, and disability. It affects approximately 1% of the adult population worldwide and is frequently associated with extra-articular manifestations involving the cardiovascular, pulmonary, ocular, and nervous systems [1]. Persistent systemic inflammation, driven by pro-inflammatory cytokines such as TNF-α, IL-1, and IL-6, may also contribute to dysfunction in sensory systems, including the auditory and vestibular organs. Several studies have reported vestibular dysfunction in patients with RA [2,3]. Reportedly, it was suggested that increased latency and amplitude asymmetry ratios in vestibular evoked myogenic potential responses were associated with impaired balance control and increased fall risk [4]. Importantly, such dysfunction may contribute to balance impairment, dizziness, and falls, thereby reducing patients’ quality of life. Although disease-modifying antirheumatic drugs (DMARDs) and biologic agents have improved the management of joint inflammation and systemic symptoms, vestibular complications remain insufficiently addressed [1]. Therefore, the development of therapeutic strategies that target not only joint destruction, but also neurological and sensory manifestations is urgently needed to achieve comprehensive disease control and improve outcomes for patients with RA.

Silicon (Si)-based agent react with water to continuously generate large amounts of hydrogen [5]. Hydrogen has anti-inflammatory and antioxidant effects and has been shown to be effective on sensory cells in the inner ear [6]. Previous reports have shown that oral administration of Si-based agent can alleviate the symptoms of various inflammatory diseases, including ulcerative colitis and interstitial pneumonia [7,8]. Therefore, it is expected that Si-based agent, which are effective in treating disorders of various organs throughout the body, may potentially be effective for RA, including vestibular dysfunction.

In our previous study, we demonstrated that mice with type II collagen-induced RA caused vestibular dysfunction. Since type II collagen is abundant in the inner ear tissue, it has been suggested that the autoimmune system reacts to this type II collagen, destroying vestibular hair cells and resulting in a decline in vestibular function [9]. In this study, we used a mouse model with type II collagen-induced RA to examine whether Si-based agent could be effective in treating RA and vestibular dysfunction.

## Materials & Methods

### Ethics statement

All experiments involving mice were performed in accordance with protocols approved by the Committee of Animal Experiments of Osaka University (Approval No. 02-001-015) and adhered to the National Institutes of Health Guide for the Care and Use of Laboratory Animals. This study complied with the ARRIVE guidelines. Efforts were made to minimize the number of animals used and to reduce animal suffering. Animals showing signs of distress or reaching predefined humane endpoints were euthanized by intraperitoneal administration of pentobarbital (200 mg/kg).

### Animals and model preparation

The experiments were conducted using 7-week-old male DBA/1J mice (Japan SLC, Shizuoka, Japan). The mice were provided with rodent diet and water ad libitum, maintained at a consistent temperature of 23–25°C. The Si-based agent was prepared using the bead-milling method according to a previous report [10], mixed with AIN93M (Oriental Yeast Co., Ltd., Tokyo, Japan) at a concentration of 2.5%, and administered to mice. The group treated with the Si-based agent was designated as the Si group, and the group administered AIN93M (Oriental Yeast Co., Ltd.) was designated as the Con group. The mice were randomly divided into two groups (n = 9 per group). For reference in the measurement of serum type II collagen-specific autoantibodies, seven untreated normal 19-week-old DBA/1J mice were also prepared.

The RA mouse models were prepared by Japan SLC using an established protocol based on previous reports [9]. The RA model was established as follows: Equal volumes of an antigen solution (0.01 M acetate phosphate buffer containing 8 mg/mL bovine type II collagen) and an adjuvant solution (a mixture containing dead *Mycobacterium tuberculosis* H37Ra and 4 mg/mL Freund’s incomplete adjuvant) were placed in separate luer lock glass syringes. The antigen solution was then transferred to a syringe containing the adjuvant solution. The contents were alternately pushed between syringes and mixed to form an emulsion. The collagen solution was intradermally administered twice under isoflurane inhalation anaesthesia. The first sensitisation was performed intradermally at the base of the auricle at 8 weeks of age and the second was administered intradermally at the base of the tail three weeks later. For both administrations, the dosage was 0.025 mL per animal, equivalent to a total of 0.2 mg of collagen per animal.

The mice were assessed using an arthritis index based on a 4-point scale: 0 (no change), 1 (toe swelling), 2 (swelling of the toes and soles), 3 (swelling of the entire foot), 4 (severe swelling), and 5 (bone degeneration). If bone degeneration was observed, an additional point (+1) was assigned. A score of 1 or higher was considered indicative of arthritis [11]. A single evaluator, who was independent of the personnel responsible for arthritis induction and treatment, performed the arthritis scoring. The evaluator was unaware of the group assignments and individual treatment information at the time of assessment. The hind limb oedema rate was determined using the following formula:


$$\begin{array}{c} Th\text{e\textasciitilde{}hindlimb\textasciitilde{}oedema\textasciitilde{}rate\textasciitilde{}(\textbackslash{}\%)}=\text{\textasciitilde{}}\frac{\text{(Hindlimb\textasciitilde{}volume\textasciitilde{}after\textasciitilde{}treatment}-\text{\textasciitilde{}Hindlimb\textasciitilde{}volume\textasciitilde{}before\textasciitilde{}treatment)}}{Hindlimbvolumebeforetreatment}\times 100 \end{array}$$

Moreover, to examine joint pain and degeneration associated with inflammation, the muscle-nerve reflex was evaluated in the open-leg position using the hind limb extension reflex test [12].

### Vestibulo-ocular reflex test

Mice were anesthetized with ketamine (100 mg/kg) and xylazine (10 mg/kg), and local anesthesia (1% lidocaine) was applied to the scalp. A Z-shaped metal fitting was secured to the skull using dental cement to immobilize the head during recording. Experiments were performed 24 h after attachment of the fitting [13].

Based on our previous findings [9], VOR was selected because it detected differences between normal mice and RA mouse models, whereas static tilt did not. VOR was induced by horizontal sinusoidal rotation in darkness at 0.3, 1, and 2 Hz using an automatically controlled turntable [14]. Eye movements were recorded with a high-speed infrared camera at 240 Hz and synchronized with turntable movement using StreamPix software. For each frequency, 7–14 recordings were obtained, and 3–5 recordings with adequate image quality were selected for analysis.

### Eye movement analysis

Eye movements were analyzed from 645 × 485-pixel images using a previously described method [13,15]. The three-dimensional position of the pupil center was reconstructed from the two-dimensional pupil ellipse and the estimated center of eyeball rotation. Horizontal eye rotation was then calculated from the reconstructed pupil coordinates. The angular position of the turntable was determined from two markers using the atan2 function [13], and angular velocities of the turntable and eye movements were calculated by differentiating their respective angular positions.

### VOR gain calculation

Turntable and eye angular velocities were fitted to sinusoidal functions by least-squares regression. VOR gain was calculated as the ratio of peak eye velocity to peak turntable velocity [16–18].

### Tissue sample preparation and Hematoxylin-Eosin stain

Mice were fixed by perfusion using 4% paraformaldehyde (PFA) fixative under anaesthesia with a combination of anesthetic (0.3 mg/kg medetomidine, 4.0 mg/kg midazolam, and 5.0 mg/kg butorphanol) [19]. Temporal bones, including the cochlea and semicircular canal, were quickly removed under a microscope (Leica, Wetzlar, Germany) and replaced with cold phosphate-buffered saline (PBS, pH 7.4). The extracted temporal bone was immersed in a 4% PFA fixative at 4°C overnight and then immersed in 10% EDTA in PBS for a week at 4°C for decalcification. The vestibular ganglia were removed after perfusion and postfixed using the same fixative. The samples were dehydrated using a series of ascending alcohol concentrations and embedded in paraffin (tissue preparation, T580, FALMA, Tokyo, Japan) using Clear Plus (FALMA). Sections of 5 μm thickness were prepared with a Rotary microtome (Leika) and mounted on MAS-coated glass slides (Matsunami-glass, Osaka, Japan). The samples were stored at 4°C until further use. After deparaffinisation, the samples were further de-paraffinised with xylene, hydrated through a series of descending alcohol concentrations, and stained with HE solution (FUJIFILM Wako Chemicals Corporation, Osaka, Japan). All stained samples were analysed using a Keyence microscope (KEYENCE Corporation, Osaka, Japan).

### Magnetic resonance imaging (MRI)

A Gd MRI contrast agent (0.5 M gadoteridol) was administered intraperitoneally at a dose of 0.10 mL per 20 g of body weight to distinguish the scala vestibuli, scala tympani, and scala media [20]. Sagittal cochlear magnetic resonance images were acquired at 90 min (right side) or 120 min (left side) after Gd administration. *In vivo* MRI of the mice was performed using an 11.7 T vertical bore scanner (AVANCE II 500WB; Bruker BioSpin, Ettlingen, Germany). Anaesthesia was induced using 2.0% isoflurane and maintained using 1.6% isoflurane during the MRI procedure. Mouse body temperatures were kept at 37°C using circulating warm water. T1-weighted 10 sagittal images of both cochleae were obtained using Rapid Acquisition with Relaxation Enhancement technique [21]. The imaging parameters were as follows: field of view (FOV) = 20 × 20 mm, matrix size = 512 × 512, in-plane resolution = 39 μm, slice thickness = 200 μm, repetition time (TR) = 800 ms, echo time (TE) = 14.8 ms, echo train length = 4, number of averages (NA) = 16, and acquisition time (TA) for each cochlea = 27 min.

### Evaluation of the endolymphatic space using inner ear MRI

The endolymphatic space was evaluated using the method described by Imai et al [22]. Briefly, inner ear MRI images at the level of the maximum cochlear axis were magnified 15-fold, and the boundaries of the endolymphatic and perilymphatic spaces were delineated.

The numbers of pixels within each delineated area was calculated, and the area ratio of the endolymphatic space to the perilymphatic space was determined. The area ratio was compared between the Con and Si groups.

### Measurement of serum type II collagen autoantibodies

Blood samples were collected from normal mice and mice from the other four groups under deep anesthesia induced with a three-drug anesthetic mixture. Serum was separated by centrifugation (3,000 rpm, 10 min, 4°C) and stored at −80°C. Type II collagen-specific autoantibodies were measured using a type II collagen antibody detection kit (Collagen Research Center, Tokyo, Japan), according to a previous report [9] and the manufacturer’s instructions. Absorbance was measured at 450 nm.

For quantitative analysis, the mean optical density (OD) of the normal mice was set to 1.0, and values for the other four groups were expressed relative to the normal mice.

### Statistical analysis

Data are presented as the mean ± SEM unless otherwise indicated. Statistical analyses were performed using Statcel 4 (Excel statistical software) and JMP® Student Edition 19.1.3. For the arthritis index and hindlimb oedema rate, linear mixed-effects model analyses were performed, with treatment group and week as fixed effects and individual mice as a random effect. When a significant treatment group × week interaction was detected, subsequent between-group comparisons at each time point were performed using Student’s t-test, with adjustment for multiple comparisons. Bone degeneration, arthritis severity (arthritis index ≥4), and leg closure rate were compared between groups using Fisher’s exact test. The duration of severe arthritis, defined as the number of weeks during which the arthritis index was ≥ 4, was compared between groups using the Wilcoxon rank-sum test. VOR data were analysed using a two-way mixed-effects model, with treatment group and stimulation frequency as fixed effects and individual animals as a random effect. The endolymphatic space area ratio measured by MRI was analysed using the Mann–Whitney U test, and serum type II collagen-specific autoantibody levels were analysed using one-way analysis of variance (ANOVA) followed by Tukey’s multiple-comparison test. A p-value of < 0.05 was considered statistically significant.

## Results

### Si-based agent showed efficacy against type II collagen-induced arthritis

First, we investigated whether an Si-based agent is effective against the symptoms of arthritis in a collagen-induced RA mouse model. Arthritis severity was assessed weekly for eight weeks after the second sensitisation using the arthritis index. In the untreated (Con) group, the arthritis index gradually increased from the 1^st^ week, peaked at the 6^th^ week, and then decreased slightly at the 8^th^ week (Fig 1a). However, the arthritis index in the Si-based agent-treated (Si) group showed a similar pattern to that in the Con group, although it remained lower in the Si group from weeks 3–8. A linear mixed-effects model revealed a significant effect of week (p < 0.0001) and a significant treatment group × week interaction (p = 0.0207), whereas the main effect of treatment group was not significant (p = 0.2476).

**Fig 1:**
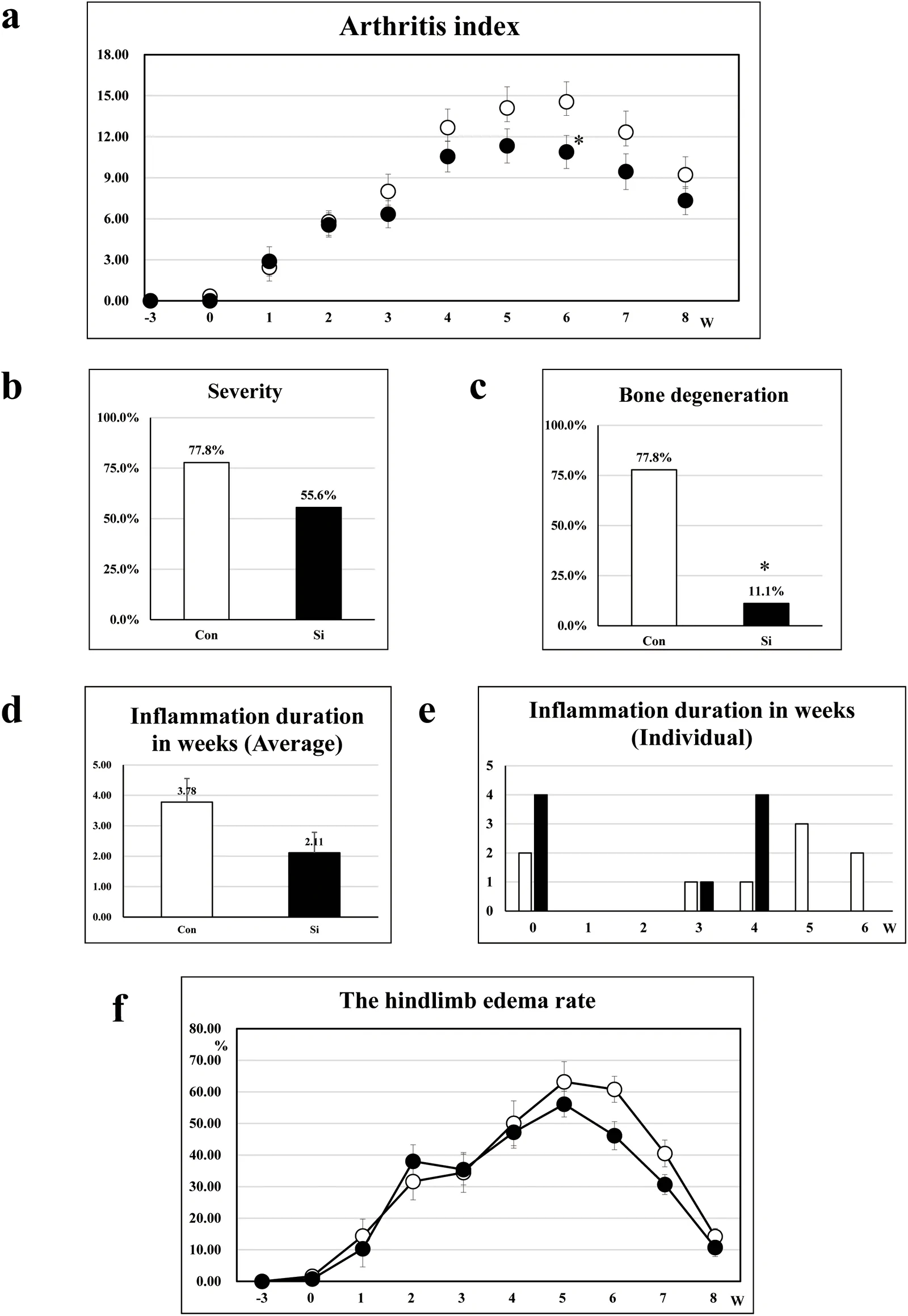
Analysis of the inflammation in type Ⅱ collagen-sensitized RA mouse models. (a) Line graph of the average arthritis index in the Con (white) and Si groups (black). X-axis notation: −3 (3 weeks before 2^nd^ sensitisation), 0 (on the day of 2^nd^ sensitisation) and 1–8 (1–8 weeks after the second sensitisation). Data are expressed as the mean ± SEM; n = 9 mice per group. Data were analysed using a linear mixed-effects model, with treatment group and week as fixed effects and individual mice as a random effect. A significant main effect of week (p < 0.0001) and a significant treatment group × week interaction (p = 0.0207) were detected, whereas the main effect of treatment group was not significant (p = 0.2476). Based on significant interaction, post hoc between-group comparisons were performed with adjustment for multiple comparisons. A significant difference between the groups was detected only at week 6 (adjusted p = 0.028). (b) Bar graph showing the proportion of mice with an arthritis index of 4 or higher in the Con (white: 77.8%) and Si (black: 55.6%) group (n = 9 mice per group). No statistically significant difference was detected between the groups (Fisher’s exact test, p = 0.6199). (c) Bar graph showing the proportion of mice with bone degeneration (arthritis index = 5) in Con (white: 77.8%) or Si (black: 11.1%) group (n = 9 mice per group). The incidence of bone degeneration was significantly lower in the Si group than in the Con group (Fisher’s exact test, p = 0.0152). (d) Bar graph showing the mean duration (in weeks) for which the arthritis index was 4 or higher in the control (white) or Si group (black). Data are expressed as the mean ± SEM; n = 9 mice per group. The duration was compared between groups using the Wilcoxon rank-sum test (p = 0.0753). (e) Bar graph of the number of mice per week that arthritis index of 4 or higher persisted in Con (white) or Si group (black). (f) Line graph of the average hindlimb oedema rate. X-axis notation: −3 (3 weeks before 2^nd^ sensitisation), 0 (on the day of 2^nd^ sensitisation) and 1–8 (1–8 weeks after the second sensitisation). Data are expressed as the mean ± SEM; n = 9 mice per group. Data were analysed using a linear mixed-effects model, with treatment group, week, and limb as fixed effects and individual mouse as a random effect. A significant main effect of week was detected (p < 0.0001), whereas no significant main effect of treatment group (p = 0.5494) or group × week interaction (p = 0.1952) was observed.

Post hoc between-group comparisons with adjustment for multiple comparisons revealed a significant difference only at week 6 (adjusted p = 0.028). Additionally, the analysis of the severity of arthritis (score of 4 or more in one of the four limbs) showed that the proportion of mice with severe arthritis in the RA mouse model was lower in the Si group than in the control group (Fig 1b, Con:77.8%, Si:55.6%), although the difference was not statistically significant (Fisher’s exact test, p = 0.6199). Bone degeneration can occur as arthritis becomes more severe [23]. When the incidence of bone degeneration was analysed, 7 of 9 mice (77.8%) in the Con group and 1 of 9 mice (11.1%) in the Si group showed bone degeneration, indicating a significantly lower incidence in the Si group (Fig 1c; Fisher’s exact test, p = 0.0152). Moreover, inflammation persisted for approximately four weeks in the Con group and for approximately two weeks in the Si group (Fig 1d). The duration of severe arthritis, defined as the number of weeks during which the arthritis index was ≥ 4, was numerically shorter in the Si group than in the Con group; however, the difference was not statistically significant (Wilcoxon rank-sum test, p = 0.0753). Particularly, inflammation persisted for more than five weeks in most of the Con group, whereas no mice in the Si group had an arthritis index ≥4 for more than five weeks (Fig 1e). Furthermore, a comparative analysis was conducted on the rate of change in limb volume of oedema associated with inflammation. Oedema was observed in both groups from the 1^st^ week, gradually increasing and peaking at the 5^th^ week. Thereafter, the oedema rate decreased (Fig 1f). The oedema rate in the Si group was lower than that in the Con group at weeks 6 and 7, although no significant difference in the time course of oedema was detected between the groups by linear mixed-effects model analysis.

Moreover, arthritis-related leg closure was assessed using the tail suspension test in mice maintained for an extended period after arthritis onset. When normal mice are suspended in the tail, they instinctively spread their legs owing to neuromuscular reflexes. Spreading the legs maintains the body position and reduces the burden on the tail by redistributing the body weight. In the Con group, most mice were unable to spread their legs when suspended by the tail because of joint pain, and their legs remained rigidly attached to the body (Fig 2a). Conversely, most mice in the Si group opened their legs as soon as their tails were suspended, and actively moved their legs. The percentage of mice that could not open their legs due to joint pain was 77.8% in the Con group and 22.2% in the Si group, indicating that arthritis-related closed legs were alleviated in the Si group (Fig 2b). Although this proportion was lower in the Si group, the difference between groups did not reach statistical significance (Fisher’s exact test, p = 0.0567).

**Fig 2:**
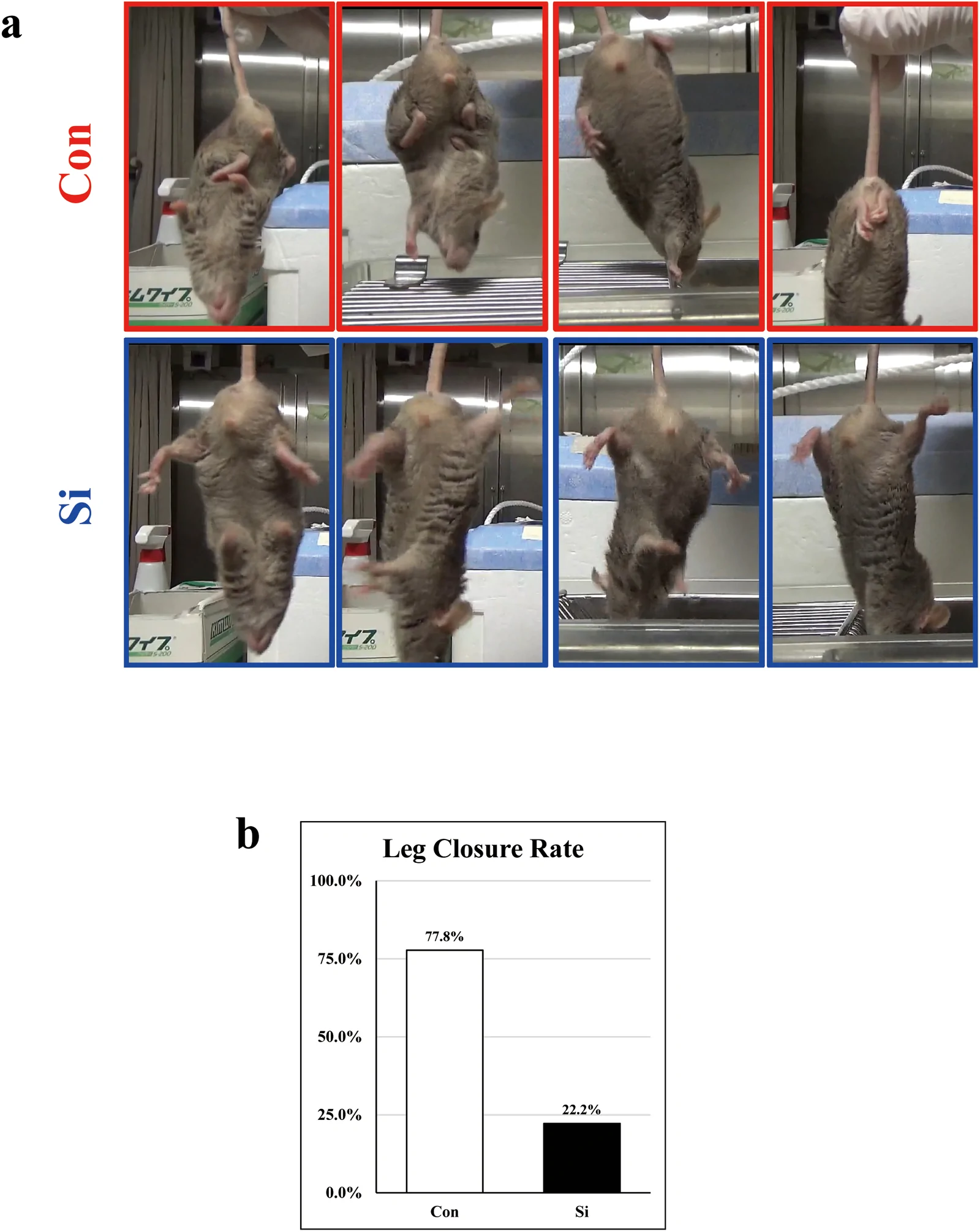
Analysis for the symptoms in type Ⅱ collagen-sensitized RA mouse models. (a) Representative images of the tail suspension test in the Con (upper line) and Si groups (bottom line). (b) The bar graph indicates the percentage of mice that could not spread their legs in the control (white, 77.8%) and Si groups (black, 22.2%). n = 9 mice per group. The difference between groups was not statistically significant (Fisher’s exact test, p = 0.0567).

Taken together, these findings suggest that Si-based agent may alleviate joint pain by reducing the inflammation associated with arthritis.

### No significant differences in VOR gain between the Con and Si groups

Next, VOR analysis was performed to examine whether the Si-based agent affected VOR-related vestibular function in RA mouse models. Lateral semicircular canal function was evaluated using the VOR gain when three stimulation patterns (0.3, 1.0, and 2.0 Hz) were applied. The VOR gain in the Con group was 0.60 ± 0.038 at 0.3 Hz, 0.61 ± 0.039 at 1.0 Hz, and 0.61 ± 0.045 at 2.0 Hz (Fig 3). The VOR gain in the Si group was 0.63 ± 0.034 at 0.3 Hz, 0.60 ± 0.019 at 1.0 Hz, and 0.64 ± 0.058 at 2.0 Hz (Fig 3).

**Fig 3:**
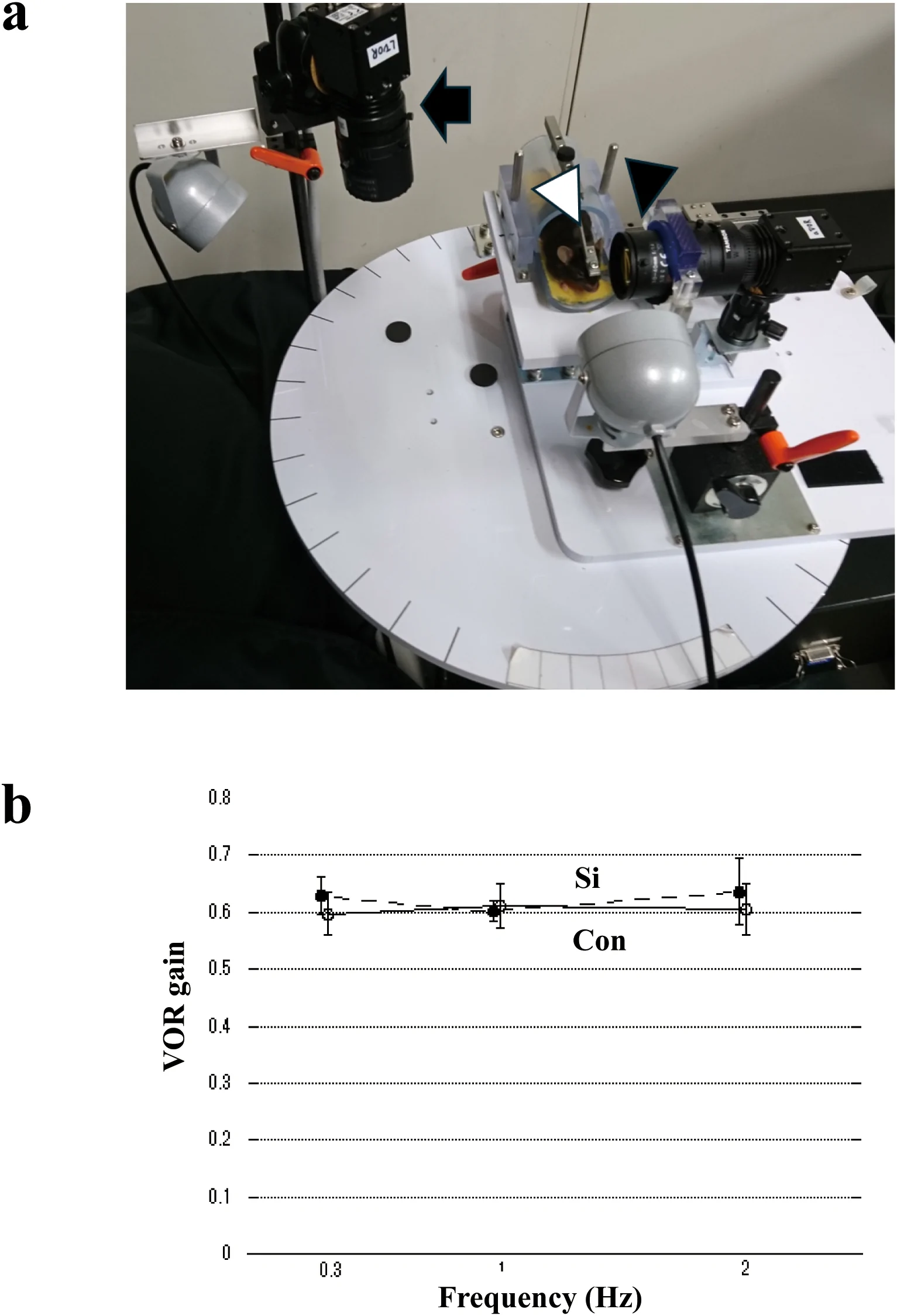
VOR analysis results for RA mouse models. (a) Overview of VOR equipment. Arrowhead: Mouse fixture. High-speed (240 Hz) infrared camera for capturing the movement of the mouse’s left eyeball (black arrowhead) and movement of the rotating device (black arrow). (b) Line graph of the mean VOR in the Con (white circle) and Si groups (black circle) when stimulated at 0.3, 1, and 2 Hz. Data are expressed as the mean ± SEM; n = 9 mice per group. Data were analyzed using a two-way mixed-effects model, with treatment group and stimulation frequency as fixed effects and individual animals as a random effect. No significant main effect of treatment group (p = 0.4927), stimulation frequency (p = 0.9325), or group × frequency interaction (p = 0.8629) was detected.

A two-way mixed-effects model, with treatment group and stimulation frequency as fixed effects and individual animals as a random effect, showed no significant main effect of treatment group (p = 0.4927) or stimulation frequency (p = 0.9325). No significant group × frequency interaction was detected (p = 0.8629).

Taken together, no significant differences in VOR gain between the Si and Con groups was detected across the stimulation frequencies examined.

### No significant differences in inner ear histological findings between the Con and Si groups

Subsequently, to determine whether the Si-based agent could alleviate the structural abnormalities in the inner ear structure in the RA mouse model, the structures of the crista ampullaris of the semicircular canal, otolith organs (utricle and saccule), and vestibular ganglion were evaluated using HE staining. Vacuolisation of the hair cell cytoplasm and loss of the cell nucleus were observed in the crista ampullaris of the semicircular canals and otolith organs in both groups. However, there was no significant difference in the degree of structural collapse between the two groups (Fig 4). In the vestibular ganglion, some areas of the ganglion cells were lost in both groups; however, no significant differences were observed between the two groups (Fig 4f).

**Fig 4:**
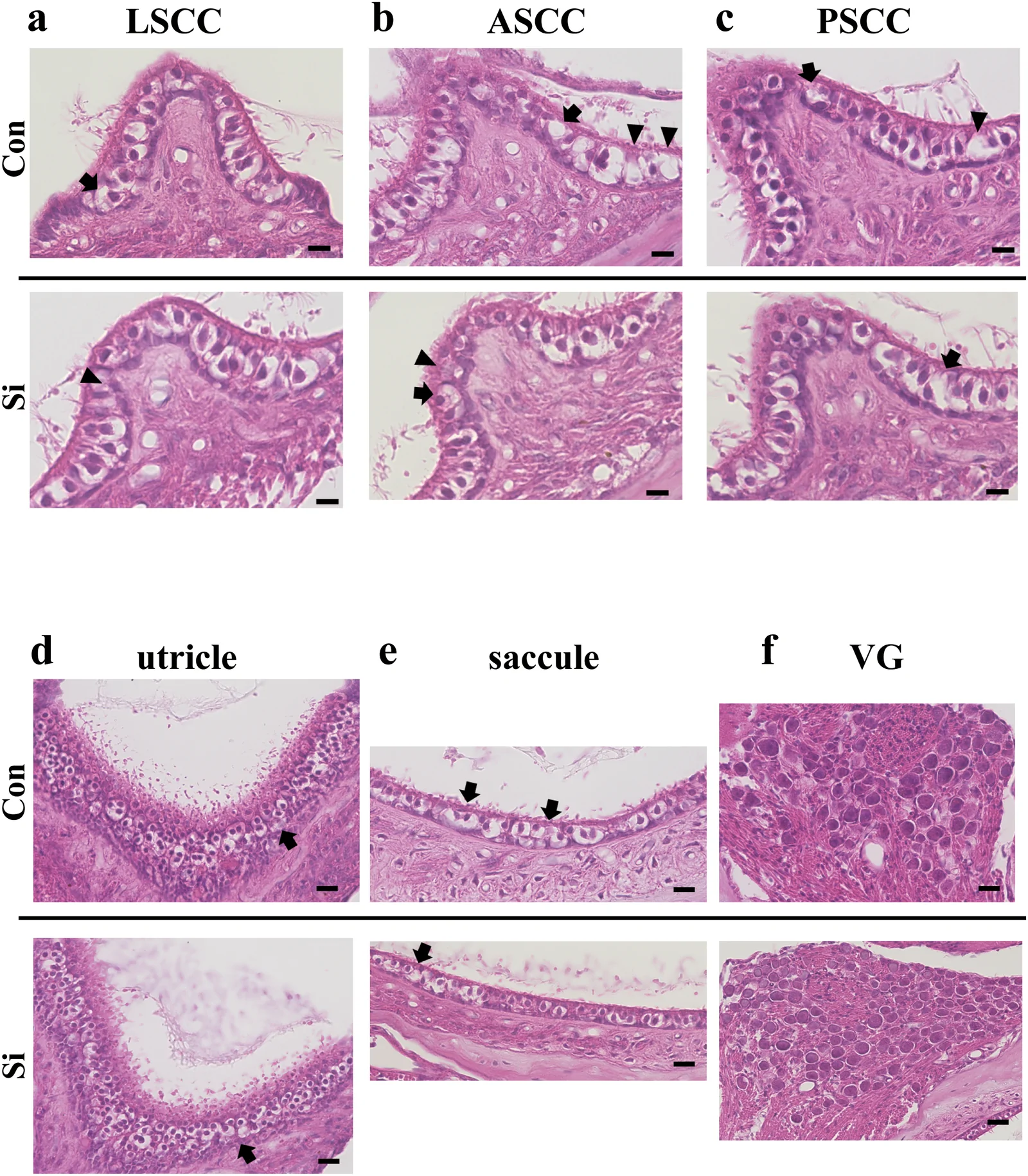
Pathological analysis using HE-stained samples. (a-f) Representative examples of HE-stained images of the lateral semicircular canal (a), the anterior semicircular canal (b), the posterior semicircular canal (c), the utricle (d), the saccule (e) and the vestibular ganglion (f) in the Con (upper images; nine mice) and the Si group (bottom lines; nine mice). Cellular vacuolisation (arrows) and destructed hair cells (arrows). (f) No degenerated and atrophied vestibular ganglion cells of both groups. LSCC: lateral semicircular canal. ASCC: anterior semicircular canal. PSCC: posterior semicircular canal. VG; vestibular ganglion. Scale bar: 20 µm.

Taken together, no significant differences in the histological findings of the inner ear were detected between the Con and Si groups.

### No significant difference in the endolymphatic space area ratio between groups

Our previous study revealed that the stria vascularis and spiral ligament were structurally disrupted in RA model mice [9]. These areas are closely involved in endolymph homeostasis. We therefore examined whether long-term administration of the Si-based agent was associated with changes in the endolymphatic space in RA mouse models. To assess the endolymphatic space, MRI analysis of the inner ear was performed in the Si and Con groups.

The image of the area where the modiolus was at its maximum was enlarged by 1500% (Fig 5a and 5b). The area ratio of the endolymphatic space (within the red frame) to the perilymphatic space (within the yellow frame) was calculated and compared between the Si and control groups. The area ratios were 48.6% (SEM ± 2.1%) in the Con group and 52.7% (SEM ± 2.0%) in the Si group (Fig 5c). No significant difference was observed between the two groups (p = 0.1138).

**Fig 5:**
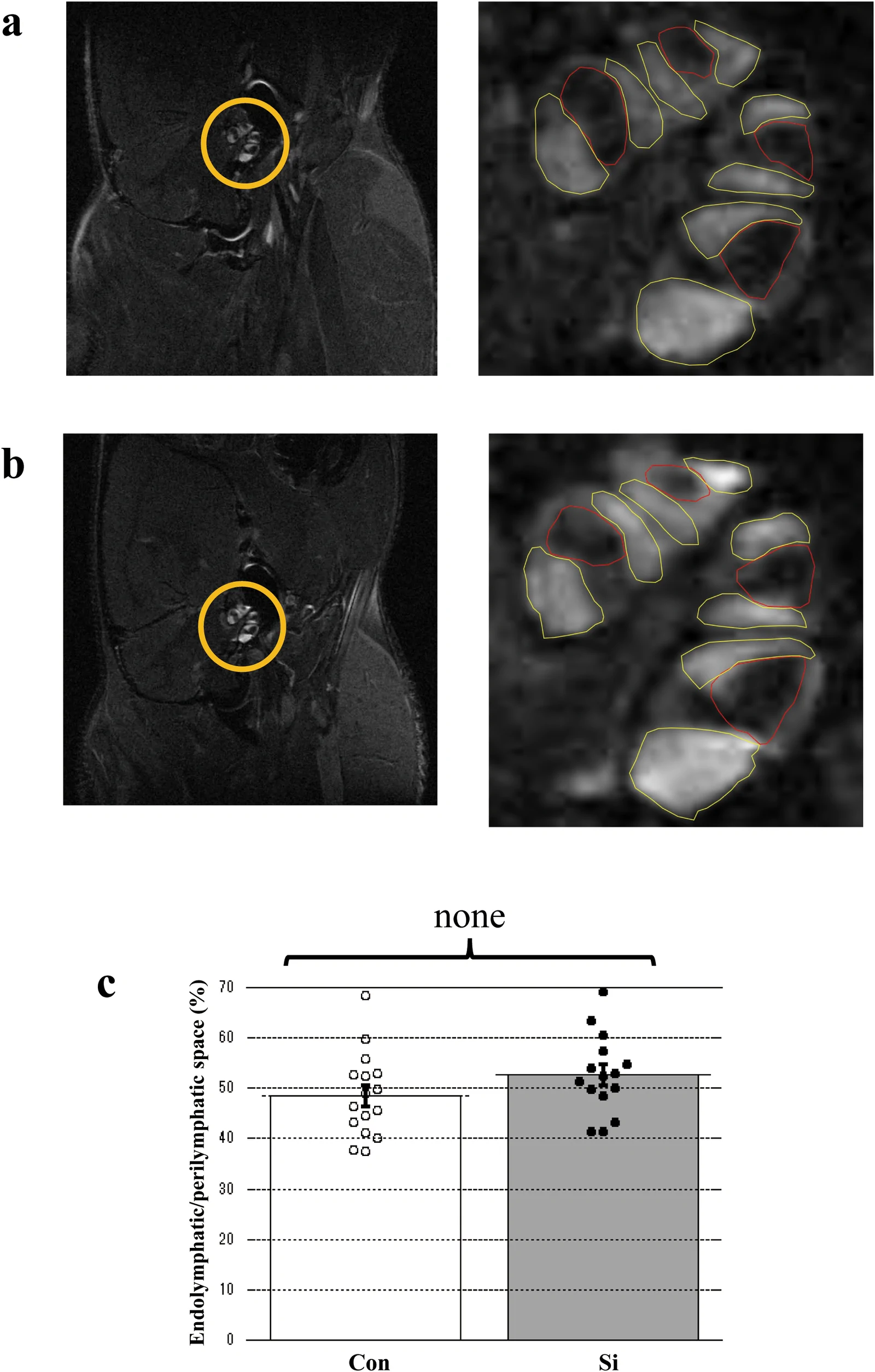
Evaluation of endolymphatic space using MRI analysis. (a, b) Representative examples of MRI images in the Con (a) and the Si group (b). The left photo is the actual size, and the right photo is a 15× image of orange circle for evaluation. Yellow and red circles indicate the perilymphatic and endolymphatic space, respectively. (c) Bar graph showing the ratio of the endolymphatic/perilymphatic space. To evaluate endolymphatic space, the ratio (%) of the endolymphatic/perilymphatic space pixel count was calculated. Data are expressed as the mean ± SEM of nine mice per group. None: no significance.

Taken together, no statistically significant difference in the endolymphatic space area ratio was detected between the Si and Con groups.

### Si-based agent attenuated the dramatic increase in type II collagen autoantibodies associated with RA

Finally, we examined whether the Si-based agent affected blood levels of type II collagen autoantibodies. ELISA showed that 8 weeks after sensitisation, the autoantibody level in the Con group was significantly increased by more than 18 times compared to that in the normal group (p < 0.0001) (Fig 6). Conversely, autoantibody levels in the Si group was significantly lower than that in the Con group (p = 0.0016), although it remained significantly higher than that in the Normal group (p = 0.0148). In contrast, the autoantibody levels in the ConL and SiL groups did not differ significantly from those in the Normal group. Normal mice showed the same levels as the negative controls without serum samples, with no detectable autoantibodies.

**Fig 6:**
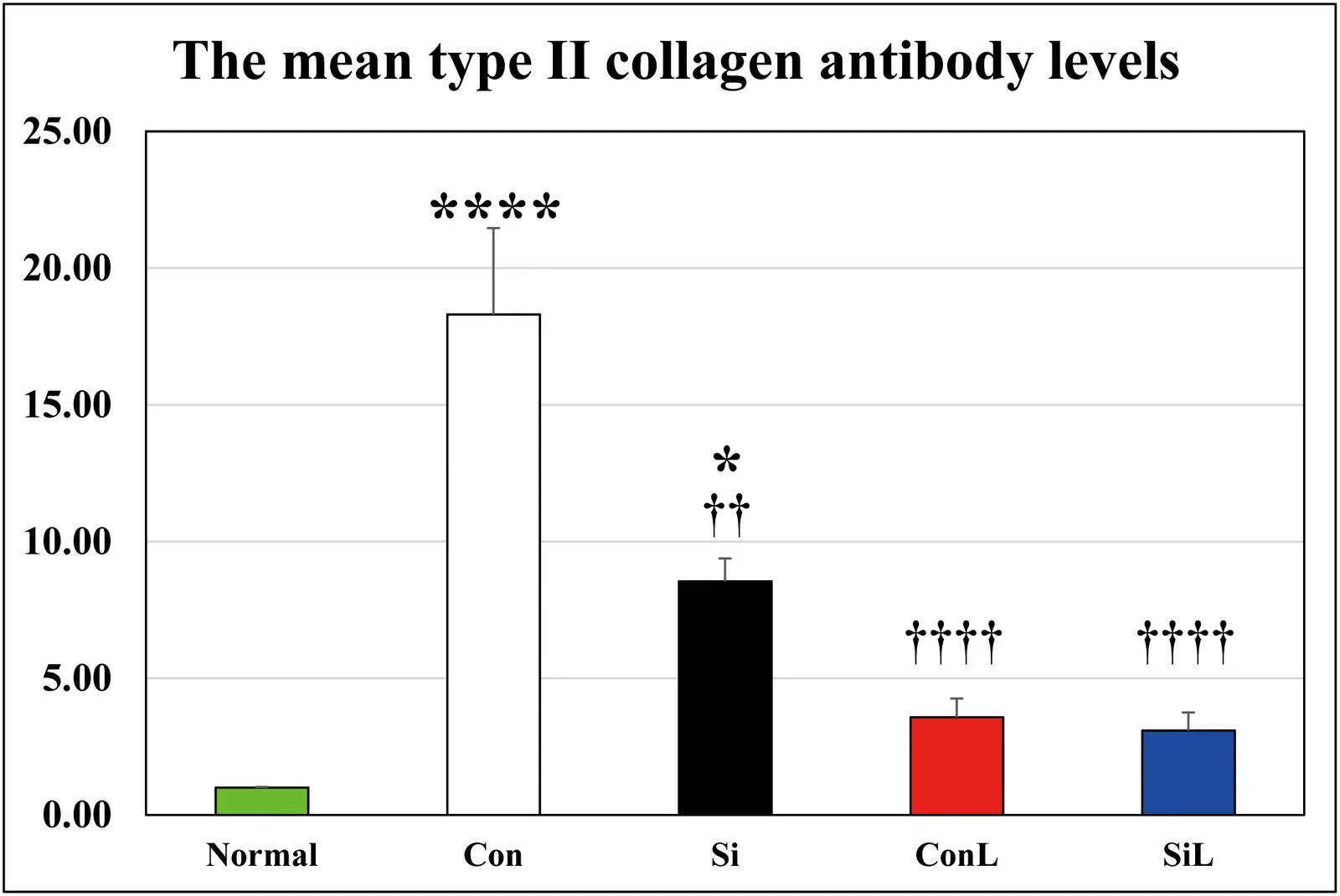
ELISA for detection type II collagen autoantibody. The bar chart indicates mean type II collagen antibody levels in the normal (green), control (white), Si (black), ConL (red), and SiL (blue) groups. Data are expressed as the mean ± SEM of nine mice per group. ConL: control group at 40 weeks after sensitisation; SiL: Si group at 40 weeks after sensitisation. One-way ANOVA showed a significant difference among the groups (p < 0.0001), followed by Tukey’s multiple-comparison test. ^****^p < 0.0001 and ^*^p < 0.05 vs. the Normal group; ^††††^p < 0.0001 and ^††^p < 0.01 vs. the Con group.

Furthermore, the amount of autoantibodies in the blood of each group, ConL and SiL groups, was also examined 40 weeks after sensitization. The autoantibody levels in both groups were significantly lower than that in the Con group (ConL, p < 0.0001; SiL, p < 0.0001), whereas no significant differences were observed between either long-term group and the Normal group. One-way ANOVA followed by Tukey’s multiple-comparison test was used for statistical analysis.

Taken together, these findings suggest that the Si-based agent was associated with a significant attenuation of the increase in type II collagen autoantibodies observed 8 weeks after sensitisation.

## Discussion

In the present study, the Si-based agent showed beneficial effects on selected arthritis-related outcomes in a type II collagen-induced arthritis model. The Si group showed a lower arthritis index at week 6 and a lower incidence of bone degeneration. However, differences in severe arthritis, leg closure, and the duration of severe arthritis were not statistically significant. In addition, serum autoantibody levels were suppressed at the earlier assessment. In contrast, no significant differences in VOR or inner-ear structural outcomes were detected between the Si-based agent and control groups. These findings suggest that the beneficial effects of the Si-based agent on arthritis were not accompanied by detectable changes in the vestibular outcomes examined in this study. Further investigation is needed to clarify the mechanisms underlying RA-associated vestibular dysfunction and potential therapeutic strategies for such complications.

The main symptoms of RA, an autoimmune disease, are joint stiffness, swelling, and pain. As inflammation persists, joint structures are damaged [24]. Therefore, anti-inflammatory agents are essential in RA treatment. Methotrexate (MTX), the first-line drug for RA, promotes adenosine release, reducing inflammation [25]. NSAIDs are commonly combined with MTX [26], but long-term use raises concerns about gastrointestinal side effects [27]. Thus, safer anti-inflammatory strategies are needed.

Si-based agent exerts anti-inflammatory effects through hydrogen produced upon reaction with water [28]. Hydrogen suppresses pro-inflammatory factors such as IL-6, IL-1β, TNF-α, and ROS. We previously showed that oral Si-based agent alleviates inflammatory disease symptoms, including ulcerative colitis and interstitial pneumonia [7,8]. Furthermore, Si-based agent increases reactive sulfur species (RSS), a strong endogenous anti-inflammatory mediator [29]. These mechanisms collectively suggest strong anti-inflammatory potential. The Si-based agent alleviated inflammation in type II collagen-induced RA mouse models and significantly suppressed collagen autoantibody levels (Fig 1, 6). Because autoantibodies drive abnormal immune activation [30], their reduction is important in RA therapy. The suppression of autoantibody production may contribute to the beneficial effects of the Si-based agent on RA-related manifestations. However, the mechanism by which Si-based agent may suppress B-cell activity remains unclear. Since no side effects have been reported, Si-based agent may serve as a potentially safe therapeutic option for RA.

Our previous work showed that type II collagen-induced RA mouse models were associated with hearing loss and vestibular dysfunction [9]. Autoantibodies against type II collagen may promote CD4 ⁺ T-cell–mediated macrophage activation and cytokine production, causing tissue damage [31]. Because type II collagen is present in the inner ear, autoantibody-mediated tissue injury may contribute to vestibular dysfunction. Although Si-based agent has anti-inflammatory effects in various organs, no significant differences in VOR gain or inner-ear structural outcomes were detected between the Si and Con groups in the present study (Fig 3, 4). Comparisons with our previous findings in 19-week-old RA mice [9] also showed no clear difference in VOR gain or inner-ear morphology.

Interestingly, no further changes in the VOR-related outcomes were detected during the later stage of arthritis in the present study. In contrast, RA severity worsens over time, including joint damage and bone mineral loss [23]. This discrepancy may reflect distinct temporal patterns of arthritis-related and vestibular changes. RA severity correlates with autoantibody levels and exposure duration [32]. Although autoantibody levels were high at 8 weeks and later declined at 48 weeks (Fig 6), vestibular function did not recover, raising the possibility that vestibular dysfunction may persist after early disease development. The Si-based agent alleviated chronic RA symptoms, such as reduced joint flexibility observed in tail suspension testing, consistent with chronic RA progression.

These findings suggest that early disease-related changes may contribute to subsequent vestibular dysfunction. No immune cell infiltration was observed in the inner ear in either 19-week or long-term mice [9]. Because arthritis onset generally occurs within several weeks after the second sensitisation, early immune-mediated changes may occur during this period. Although autoantibodies are reportedly produced equally in acute and chronic stages [33], we observed long-term reductions in the Si group; however, group differences disappeared, suggesting that the suppressive effect of the Si-based agent on autoantibody levels may be limited at later stages. We hypothesize that vestibular injury may occur when autoantibody levels reach a threshold sufficient to promote inner-ear entry. Further studies correlating serum autoantibody levels with vestibular onset may provide therapeutic insights.

MRI analysis showed increased endolymphatic space area ratios in both groups compared with 19-week-old mice [9]. Tissue destruction and atrophy in the cochlear stria vascularis were observed (Fig 4), similar to findings from earlier stages. Since endolymph is produced by the stria vascularis, its degeneration may contribute to alterations in endolymphatic homeostasis. Similar long-term enlargement of the endolymphatic space has been reported in guinea pig RA models [34]. These findings suggest that long-term CIA may be associated with persistent structural changes in the inner ear, which may be relevant to vestibular dysfunction and should be considered when evaluating therapeutic interventions at later stages.

A limitation of the present study is the absence of a normal control group. Therefore, we could not determine whether vestibular dysfunction was reproduced in the present type II collagen-induced RA mouse model. In our previous study [9], VOR gain was reduced only at 2 Hz, and the magnitude of the difference was relatively small. Thus, the present vestibular findings should be interpreted cautiously. Although MRI revealed changes in the endolymphatic space in the long-term RA mouse model, the absence of a normal control group also limited our ability to determine whether these changes were specifically associated with RA. In addition, the sample size was not determined based on a formal a priori power analysis; therefore, the possibility of insufficient statistical power and a type II error cannot be excluded, particularly for the negative findings. Accordingly, the absence of statistically significant differences between the Si and Con groups should be interpreted as a lack of detectable differences under the conditions of the present study, rather than evidence that the Si-based agent has no effect on vestibular or inner-ear outcomes. Further studies with an appropriate normal control group, adequately determined sample sizes, and longitudinal assessments are warranted to clarify the relationship between RA, vestibular dysfunction, and changes in the inner ear.

In summary, the present findings suggest that the therapeutic effects of the Si-based agent may differ according to the pathological manifestations of RA. The beneficial effects observed on arthritis-related outcomes may have implications for the development of therapeutic approaches aimed at improving quality of life, while the mechanisms underlying RA-associated vestibular dysfunction and inner-ear structural changes remain to be elucidated. Further studies are warranted to clarify these mechanisms and explore therapeutic strategies for vestibular complications of RA.

## Supporting information

S1 Table The Excel files labeled “Arthritis Index,” each containing raw data for individual mice regarding the arthritis index. (XLSX)

S2 Table The Excel files labeled “edema rate,” each containing raw data for individual mice regarding the edema rate. (XLSX)

S3 Table The Excel files labeled “Autoantibody levels,” each containing raw data for individual mice regarding the autoantibody levels. (XLSX)

S4 Table The Excel files labeled “VOR,” each containing raw data for individual mice regarding the VOR. (XLSX)

S5 Table Author checklist of ARRIVE Guideline. (PDF)
