# Supplementary material for: Evaluation of the efficacy of Si-based agent in Rheumatoid Arthritis with complicating vestibular impairment
Source: PLoS One. 2026 Nov 6;21(11):e0361954. doi: 10.1371/journal.pone.0361954 (PMC13742939; doi:10.1371/journal.pone.0361954)
Supplement: Supplementary file 3 [file pone.0361954.s008.docx]

**Editor’s comment
Thank you for submitting your manuscript to PLOS One. After careful consideration, we feel that it has merit but does not fully meet PLOS One’s publication criteria as it currently stands. Therefore, we invite you to submit a revised version of the manuscript that addresses the points raised during the review process.
Response**
Thank you for the opportunity to revise our manuscript. We have carefully addressed the reviewers’ comments and revised the manuscript accordingly.

**Reviewer #1: The authors have adequately addressed most of my previous comments, and the manuscript has improved substantially. However, a few issues remain.**

**Response**

Thank you for your positive assessment of our revised manuscript. We have carefully addressed the remaining comments and revised the manuscript accordingly.

**Major comments
1. Multiple comparisons (Fig. 1a)
The use of a linear mixed-effects model is an improvement. However, the subsequent Student's t-tests at individual time points were performed without adjustment for multiple comparisons. I suggest omitting these post hoc comparisons and focusing on the significant group × week interaction. Alternatively, an appropriate multiple-comparison adjustment should be applied.
Response 1**

Thank you for this important comment. The between-group comparisons at individual time points were performed based on the significant group × week interaction, with adjustment for multiple comparisons. We have clarified the statistical procedure in both the “***Results***” section (***lines 239-240 on page 14***) and the corresponding ***Fig1a legend*** to ensure consistency in the presentation of the findings.

**2. Statistical analysis of arthritis-related outcomes (Figs. 1 and 2)
The Methods section states that unpaired Student's t-tests were used for bone degeneration, arthritis severity, duration of inflammation, and leg closure rate, but the corresponding p-values are not clearly reported. Since bone degeneration, arthritis severity, and leg closure rate are binary outcomes, Fisher's exact test would be more appropriate, particularly given the small sample size (n = 9 per group). Please revise the analyses accordingly and report the results. Even if some comparisons are not statistically significant, the observed differences between groups should be described and interpreted cautiously.
Response 2**

Thank you for this valuable suggestion. We agree that Fisher’s exact test is more appropriate than the unpaired Student’s *t*-test for comparing binary outcomes, particularly given the small sample size. Accordingly, we reanalyzed bone degeneration, arthritis severity, and arthritis-related leg closure using **Fisher’s exact test**. The incidence of bone degeneration was significantly lower in the Si group than in the Con group (**77.8% vs. 11.1%, *p* = 0.0152**). In contrast, the differences in the proportion of mice with severe arthritis (**77.8% vs. 55.6%, *p* = 0.6199**) and arthritis-related leg closure (**77.8% vs. 22.2%, *p* = 0.0567**) were not statistically significant. The duration of severe arthritis, defined as the number of weeks during which the arthritis index was ≥4, was compared using the Wilcoxon rank-sum test. Although this duration was numerically shorter in the Si group, the difference was not statistically significant (***p* = 0.0753**).

We have revised the “***Statistical Analysis***” subsection of the “***Materials & Methods***” (***lines 212, 216-220 on page 13***) and “***Results***” (***lines 243-254 on page 15, 261-262, lines 270-273 on page 16***), “***Discussion***” section (***lines 341-345 on page 20***), and **Fig.1,2 legends** to reflect the updated statistical analyses and results. We have also revised the interpretation to distinguish statistically significant findings from numerical differences that did not reach statistical significance.

**3. Interpretation of the findings
Although the Discussion has been revised appropriately, some statements in the Results remain overly definitive. For example, the heading at lines 279–280 and the concluding statement at lines 291–293 imply that the Si-based agent was ineffective. However, the absence of detectable differences does not establish the absence of a treatment effect. Furthermore, the qualitative histological findings do not directly support conclusions about vestibular function. Please revise these statements and ensure consistent interpretation throughout the manuscript.
Response 3**

Thank you for this valuable comment. We have revised the Results section to avoid overly definitive statements regarding the effects of the Si-based agent. Specifically, we have revised the relevant headings and concluding statements to clarify that the absence of statistically significant differences does not establish the absence of a treatment effect. We have also revised the description of the histological findings to limit the conclusions to the observed structural changes, without inferring effects on vestibular function. These revisions have been made consistently throughout the “***Results***” section (***line 276 on page 16, lines 290-293 on page 17, lines 301-302, 307-309, 316-317 on page 18***).

**Minor comments
1. Line 248: “Fig. 1g” should be corrected to “Fig. 1f”.
Response 1**

Thank you for pointing this out. We have corrected “***Fig. 1g***” to “***Fig. 1f***” in ***Line 257*** of the revised manuscript.

**2. Lines 569–572: The descriptions of Figures 1b and 1c appear to be reversed. Please correct the figure legends.**

**Response 2**

Thank you for pointing this out. We have revised the figure legends to clarify that **Fig. 1b** shows the proportion of mice with **an arthritis index of 4 or higher (arthritis severity)**, whereas ***Fig. 1c*** shows the proportion of mice with **bone degeneration (arthritis index = 5)**. Because the Results section describes arthritis severity followed by bone degeneration, we have also switched the descriptions of ***Fig. 1b*** and ***Fig. 1c*** in the **Figure legends** to maintain consistency with the order of presentation in the “***Results***” section. The **Figure legends** have been corrected accordingly.
