# Supplementary material for: Evaluation of the efficacy of Si-based agent in Rheumatoid Arthritis with complicating vestibular impairment
Source: PLoS One. 2026 Nov 6;21(11):e0361954. doi: 10.1371/journal.pone.0361954 (PMC13742939; doi:10.1371/journal.pone.0361954)
Supplement: Peer review history

# Peer review: Decision Letter 0

**DOI:** 10.1371/journal.pone.0361954.r001

**Authors:** Toru Miwa

11 Sep 2026

Dear Dr. Koyama,

Thank you for submitting your manuscript to PLOS One. After careful consideration, we feel that it has merit but does not fully meet PLOS One’s publication criteria as it currently stands. Therefore, we invite you to submit a revised version of the manuscript that addresses the points raised during the review process.

Please submit your revised manuscript by Nov 10 2026 11:59PM. If you will need more time than this to complete your revisions, please reply to this message or contact the journal office at plosone@plos.org. When you're ready to submit your revision, log on to https://www.editorialmanager.com/pone/ and select the 'Submissions Needing Revision' folder to locate your manuscript file.

- A letter that responds to each point raised by the academic editor and reviewer(s). You should upload this letter as a separate file labeled 'Response to Reviewers'.
- A marked-up copy of your manuscript that highlights changes made to the original version. You should upload this as a separate file labeled 'Revised Manuscript with Track Changes'.
- An unmarked version of your revised paper without tracked changes. You should upload this as a separate file labeled 'Manuscript'.

If you would like to make changes to your financial disclosure, please include your updated statement in your cover letter. Guidelines for resubmitting your figure files are available below the reviewer comments at the end of this letter.

If applicable, we recommend that you deposit your laboratory protocols in protocols.io to enhance the reproducibility of your results. Protocols.io assigns your protocol its own identifier (DOI) so that it can be cited independently in the future. For instructions see: https://journals.plos.org/plosone/s/submission-guidelines#loc-laboratory-protocols. Additionally, PLOS One offers an option for publishing peer-reviewed Lab Protocol articles, which describe protocols hosted on protocols.io. Read more information on sharing protocols at https://plos.org/protocols?utm_medium=editorial-email&utm_source=authorletters&utm_campaign=protocols.

As the corresponding author, your ORCID iD is verified in the submission system and will appear in the published article. PLOS supports the use of ORCID, and we encourage all coauthors to register for an ORCID iD and use it as well. Please encourage your coauthors to verify their ORCID iD within the submission system before final acceptance, as unverified ORCID iDs will not appear in the published article. *Only*  the individual author can complete the verification step; PLOS staff *cannot*  verify ORCID iDs on behalf of authors.

We look forward to receiving your revised manuscript.

Kind regards,

Toru Miwa

Academic Editor

PLOS One

Journal Requirements:

When submitting your revision, we need you to address these additional requirements.

1.Please ensure that your manuscript meets PLOS ONE's style requirements, including those for file naming. The PLOS ONE style templates can be found at

https://journals.plos.org/plosone/s/file?id=wjVg/PLOSOne_formatting_sample_main_body.pdf and

https://journals.plos.org/plosone/s/file?id=ba62/PLOSOne_formatting_sample_title_authors_affiliations.pdf

2. Thank you for stating the following financial disclosure:

“This work was supported by the Center of Innovation Program (COI Program, Grant Number JPMJCE1310, JST, Japan) and JSPS KAKENHI (Grant Number 23K15862).”

Please state what role the funders took in the study.  If the funders had no role, please state: "The funders had no role in study design, data collection and analysis, decision to publish, or preparation of the manuscript."

If this statement is not correct you must amend it as needed.

Please include this amended Role of Funder statement in your cover letter; we will change the online submission form on your behalf.

3. Thank you for stating the following in the Acknowledgments Section of your manuscript:

“This research was supported by the Center of Innovation Program (COI Program, Grant Number JPMJCE1310, JST Japan, and JSPS KAKENHI (No. 23K15862). We thank Ms. M.  Hasegawa for technical assistance. We would like to thank Editage (www.editage.com) for English language editing and the Center for Medical Research and Education, Graduate School for Medicine, Osaka University, for technical support ”

We note that you have provided funding information that is not currently declared in your Funding Statement. However, funding information should not appear in the Acknowledgments section or other areas of your manuscript. We will only publish funding information present in the Funding Statement section of the online submission form.

Please remove any funding-related text from the manuscript and let us know how you would like to update your Funding Statement. Currently, your Funding Statement reads as follows:

“This work was supported by the Center of Innovation Program (COI Program, Grant Number JPMJCE1310, JST, Japan) and JSPS KAKENHI (Grant Number 23K15862).”

Please include your amended statements within your cover letter; we will change the online submission form on your behalf.

4. Your ethics statement should only appear in the Methods section of your manuscript. If your ethics statement is written in any section besides the Methods, please move it to the Methods section and delete it from any other section. Please ensure that your ethics statement is included in your manuscript, as the ethics statement entered into the online submission form will not be published alongside your manuscript.

5. Please include captions for your Supporting Information files at the end of your manuscript, and update any in-text citations to match accordingly. Please see our Supporting Information guidelines for more information: http://journals.plos.org/plosone/s/supporting-information.

6. If the reviewer comments include a recommendation to cite specific previously published works, please review and evaluate these publications to determine whether they are relevant and should be cited. There is no requirement to cite these works unless the editor has indicated otherwise.

[Note: HTML markup is below. Please do not edit.]

Reviewer's Responses to Questions

**Comments to the Author**

1. Is the manuscript technically sound, and do the data support the conclusions?

Reviewer #1: Partly

**********

2. Has the statistical analysis been performed appropriately and rigorously?

Reviewer #1: No

**********

3. Have the authors made all data underlying the findings in their manuscript fully available?

The PLOS Data policy

Reviewer #1: No

**********

4. Is the manuscript presented in an intelligible fashion and written in standard English?

Reviewer #1: Yes

**********

Reviewer #1: This study investigated the effects of a Si-based agent on arthritis and vestibular dysfunction using a type II collagen-induced arthritis (CIA) mouse model. This work extends the authors’ previous study (Ref. 9). The authors evaluated arthritis severity, VOR, histological findings, endolymphatic hydrops by MRI, and serum type II collagen antibody levels.

However, there are several important concerns regarding the study design, statistical analysis, and interpretation of the results that weaken the main conclusion that the Si-based agent improves arthritis but has no effect on vestibular dysfunction. In addition, the Methods section contains missing information, unnecessary overlap with the previous publication, and inconsistencies with the presented results. Addressing these issues would substantially strengthen the manuscript.

Major comments

1. The most important limitation of this study is the lack of a normal control group. Only untreated and treated CIA mice were compared. Therefore, it is impossible to confirm whether vestibular dysfunction was actually reproduced in the present experiment. In the previous study (Ref. 9), VOR gain was reduced only at 2 Hz, and the difference was relatively small. This important limitation should be discussed more carefully. Based on the present data, it is appropriate to conclude only that no significant difference was detected between the two CIA groups, rather than concluding that the Si-based agent is ineffective for vestibular dysfunction.

2. In the previous study, both VOR and static tilt analyses were performed, and static tilt appeared to detect vestibular dysfunction more clearly. In the present study, only VOR was evaluated, but the reason for excluding static tilt analysis is not explained. In addition, VOR mainly reflects lateral semicircular canal function. Therefore, the conclusion should not be generalized to overall vestibular function. Please explain why only VOR was selected.

3. The description of the statistical analysis is insufficient. The Methods section states that Student’s t-test or Mann-Whitney U test was used; however, it is unclear which statistical test was applied to each analysis. Please specify the statistical method used for each figure or dataset. In addition, Figure 6 includes three groups (Normal, CIA, and CIA+Si), but the statistical method used for this comparison is not described. Furthermore, the manuscript states that a paired t-test was used for comparison with the Normal group. If these groups consisted of independent animals, a paired t-test would not be appropriate. Please clarify whether this is a description error or the actual statistical method used. The statistical analysis should be described in sufficient detail to allow readers to assess the appropriateness and reproducibility of the analyses.

4. The main conclusion of this study is based on negative results (no significant differences in VOR, histology, or MRI). However, no information is provided regarding sample size justification or statistical power. Therefore, it is difficult to determine whether there is truly no treatment effect or whether the study is underpowered (Type II error). Since the main conclusion relies on negative findings, this point should be discussed. In addition, the present data indicate that no significant difference was detected; they do not prove that the treatment has no effect. The interpretation of the negative findings should therefore be more cautious throughout the Discussion.

5. Arthritis scoring is subjective and susceptible to observer bias. The manuscript does not describe whether the assessment was performed in a blinded manner or how many evaluators were involved. Please provide these methodological details.

6. MRI was used to evaluate endolymphatic hydrops. However, the previous study did not demonstrate obvious hydrops in the CIA model. Since no normal control group was included in the present study, the rationale for including MRI as a major outcome measure should be explained more clearly in the Introduction or Discussion.

Minor comments

1. Figure 6 presents ELISA data for serum type II collagen antibody levels, but no ELISA method is described in the Methods section. Please add the experimental details, including the assay kit, procedures, and analysis. In addition, normal mice are included in Figure 6, but the Methods section does not describe these animals. Please clarify how the normal controls were prepared and analyzed.

2. The description of the VOR experiment in the Methods section is largely identical to that in the previous publication. I suggest citing the previous paper and describing only the information necessary for the present study.

3. The section entitled “Eye movement recording” (around Line 171) appears to describe the static tilt experiment. However, no static tilt results are presented in this manuscript. If this section is not relevant, it should be removed.

4. The Abstract is unbalanced. Most of the Abstract is devoted to the Background, whereas the Methods and Results are too brief. The study design, evaluation methods, and main findings should be described more clearly. A structured Abstract (or a similar format) would improve readability. The major negative findings should also be clearly stated.

Overall, this study addresses an interesting topic, and the finding that the Si-based agent improved arthritis in the CIA model is valuable. However, several important limitations in the study design, statistical analysis, and interpretation of the negative findings should be addressed before the conclusions can be fully supported. In particular, the conclusions regarding vestibular dysfunction should be limited to what is supported by the present data. Revision of the Methods and Discussion would substantially improve the manuscript.

**********

what does this mean?). If published, this will include your full peer review and any attached files.

If you choose “no”, your identity will remain anonymous but your review may still be made public.

**Do you want your identity to be public for this peer review?**  For information about this choice, including consent withdrawal, please see our Privacy Policy

Reviewer #1: No

**********

[NOTE: If reviewer comments were submitted as an attachment file, they will be attached to this email and accessible via the submission site. Please log into your account, locate the manuscript record, and check for the action link "View Attachments". If this link does not appear, there are no attachment files.]

To ensure your figures meet our technical requirements, please review our figure guidelines: https://journals.plos.org/plosone/s/figures

You may also use PLOS’s free figure tool, NAAS, to help you prepare publication quality figures: https://journals.plos.org/plosone/s/figures#loc-tools-for-figure-preparation.

NAAS will assess whether your figures meet our technical requirements by comparing each figure against our figure specifications.

# Author comment: Author response to Decision Letter 1

**DOI:** 10.1371/journal.pone.0361954.r002

21 Sep 2026

Editor

Thank you for submitting your manuscript to PLOS One. After careful consideration, we feel that it has merit but does not fully meet PLOS One’s publication criteria as it currently stands. Therefore, we invite you to submit a revised version of the manuscript that addresses the points raised during the review process.

Response

We sincerely thank the Editor for the careful consideration of our manuscript and for the constructive comments. We have carefully addressed all of the points raised and revised the manuscript accordingly. We hope that the revised manuscript will now meet the publication criteria of PLOS ONE.

Journal Requirements:

When submitting your revision, we need you to address these additional requirements.

1.Please ensure that your manuscript meets PLOS ONE's style requirements, including those for file naming. The PLOS ONE style templates can be found at

https://journals.plos.org/plosone/s/file?id=wjVg/PLOSOne_formatting_sample_main_body.pdf and

https://journals.plos.org/plosone/s/file?id=ba62/PLOSOne_formatting_sample_title_authors_affiliations.pdf

Response

Thank you for your comment. We have carefully reviewed the manuscript according to the PLOS ONE style requirements and revised the manuscript formatting and file names accordingly, following the provided PLOS ONE style templates.

2. Thank you for stating the following financial disclosure:

“This work was supported by the Center of Innovation Program (COI Program, Grant Number JPMJCE1310, JST, Japan) and JSPS KAKENHI (Grant Number 23K15862).”

Please state what role the funders took in the study. If the funders had no role, please state: "The funders had no role in study design, data collection and analysis, decision to publish, or preparation of the manuscript."

If this statement is not correct you must amend it as needed.

Please include this amended Role of Funder statement in your cover letter; we will change the online submission form on your behalf.

Response

Thank you for your comment. We have revised the “Funding” section of the manuscript (lines 435-437 on page 19) to include the following Role of Funder statement and have also included this statement in the cover letter:

“The funders had no role in study design, data collection and analysis, decision to publish, or preparation of the manuscript.”

3. Thank you for stating the following in the Acknowledgments Section of your manuscript:

“This research was supported by the Center of Innovation Program (COI Program, Grant Number JPMJCE1310, JST Japan, and JSPS KAKENHI (No. 23K15862). We thank Ms. M. Hasegawa for technical assistance. We would like to thank Editage (www.editage.com) for English language editing and the Center for Medical Research and Education, Graduate School for Medicine, Osaka University, for technical support ”

We note that you have provided funding information that is not currently declared in your Funding Statement. However, funding information should not appear in the Acknowledgments section or other areas of your manuscript. We will only publish funding information present in the Funding Statement section of the online submission form.

Please remove any funding-related text from the manuscript and let us know how you would like to update your Funding Statement. Currently, your Funding Statement reads as follows:

“This work was supported by the Center of Innovation Program (COI Program, Grant Number JPMJCE1310, JST, Japan) and JSPS KAKENHI (Grant Number 23K15862).”

Please include your amended statements within your cover letter; we will change the online submission form on your behalf.

Response

Thank you for your comment. We have removed all funding-related information from the “Acknowledgments” section of the manuscript, as requested.

4. Your ethics statement should only appear in the Methods section of your manuscript. If your ethics statement is written in any section besides the Methods, please move it to the Methods section and delete it from any other section. Please ensure that your ethics statement is included in your manuscript, as the ethics statement entered into the online submission form will not be published alongside your manuscript.

Response

Thank you for your comment. We have moved the ethics statement from the “Declarations” section to the beginning of the “Materials and Methods” section, as requested.

5. Please include captions for your Supporting Information files at the end of your manuscript, and update any in-text citations to match accordingly. Please see our Supporting Information guidelines for more information: http://journals.plos.org/plosone/s/supporting-information.

Response

Thank you for your comment. This manuscript does not contain any Supporting Information files.

6. If the reviewer comments include a recommendation to cite specific previously published works, please review and evaluate these publications to determine whether they are relevant and should be cited. There is no requirement to cite these works unless the editor has indicated otherwise.

Response

Thank you for the clarification. No specific publications were recommended for citation in the reviewer comments.

Reviewer #1

This study investigated the effects of a Si-based agent on arthritis and vestibular dysfunction using a type II collagen-induced arthritis (CIA) mouse model. This work extends the authors’ previous study (Ref. 9). The authors evaluated arthritis severity, VOR, histological findings, endolymphatic hydrops by MRI, and serum type II collagen antibody levels.

However, there are several important concerns regarding the study design, statistical analysis, and interpretation of the results that weaken the main conclusion that the Si-based agent improves arthritis but has no effect on vestibular dysfunction. In addition, the Methods section contains missing information, unnecessary overlap with the previous publication, and inconsistencies with the presented results. Addressing these issues would substantially strengthen the manuscript.

Response

Thank you for the careful and constructive evaluation of our manuscript. We have carefully addressed all of the concerns regarding the study design, statistical analysis, interpretation, Methods, and consistency with our previous publication. The manuscript has been revised accordingly to clarify and strengthen the presentation of our findings and conclusions.

Major comments

1. The most important limitation of this study is the lack of a normal control group. Only untreated and treated CIA mice were compared. Therefore, it is impossible to confirm whether vestibular dysfunction was actually reproduced in the present experiment. In the previous study (Ref. 9), VOR gain was reduced only at 2 Hz, and the difference was relatively small. This important limitation should be discussed more carefully. Based on the present data, it is appropriate to conclude only that no significant difference was detected between the two CIA groups, rather than concluding that the Si-based agent is ineffective for vestibular dysfunction.

Response 1

Thank you for this important comment. We agree that the absence of a normal control group limits our ability to determine whether vestibular dysfunction was reproduced in the present CIA model. We have added this limitation to the “Discussion” section (lines 399-413 on pages 17-18), and noted that, in our previous study [Ref No. 9 in the text], the reduction in VOR gain was limited to 2 Hz and relatively small.

Accordingly, we have revised the interpretation of the vestibular findings throughout the manuscript and now state that no significant differences in vestibular function-related outcomes were detected between the Si-based agent and control groups, rather than concluding that the Si-based agent was ineffective against vestibular dysfunction.

We have also revised the “Abstract” and “Discussion” section to ensure that the conclusions are appropriately limited to the present data.

2. In the previous study, both VOR and static tilt analyses were performed, and static tilt appeared to detect vestibular dysfunction more clearly. In the present study, only VOR was evaluated, but the reason for excluding static tilt analysis is not explained. In addition, VOR mainly reflects lateral semicircular canal function. Therefore, the conclusion should not be generalized to overall vestibular function. Please explain why only VOR was selected.

Response 2

Thank you for this important comment. In our previous study [Ref No. 9 in the text], both VOR and static tilt analyses were performed. The static tilt analysis, which evaluates otolith function, showed no significant difference between the normal and RA groups, whereas the VOR analysis detected a significant difference, indicating that semicircular canal dysfunction was more evident in the RA model. Therefore, we selected VOR testing as the vestibular assessment in the present study.

We agree that VOR primarily reflects lateral semicircular canal function and that the present findings should not be generalized to overall vestibular function. We have clarified the rationale for selecting VOR in the “Vestibulo-ocular reflex test” subsection of the “Materials and Methods” section (lines 137-138 on page 7) and have revised the “Results” (lines 264-266 on page 12) “Discussion” section (lines 331-339 on page 15, lines 365-373, 375-376 on pages 16, lines 379-380 on page 17) to acknowledge this limitation and to appropriately restrict the interpretation of our findings to the vestibular function assessed by VOR.

3. The description of the statistical analysis is insufficient. The Methods section states that Student’s t-test or Mann-Whitney U test was used; however, it is unclear which statistical test was applied to each analysis. Please specify the statistical method used for each figure or dataset. In addition, Figure 6 includes three groups (Normal, CIA, and CIA+Si), but the statistical method used for this comparison is not described. Furthermore, the manuscript states that a paired t-test was used for comparison with the Normal group. If these groups consisted of independent animals, a paired t-test would not be appropriate. Please clarify whether this is a description error or the actual statistical method used. The statistical analysis should be described in sufficient detail to allow readers to assess the appropriateness and reproducibility of the analyses.

Response 3

Thank you for this important comment. We agree that the statistical methods should be described in sufficient detail for each analysis to ensure appropriate interpretation and reproducibility.

We carefully rechecked the statistical analyses and revised the “Statistical Analysis” subsection of the “Materials and Methods” section (lines 210-221 on page 10) accordingly. The arthritis index and hindlimb oedema rate were analysed using linear mixed-effects models, followed by Student’s t-tests for between-group comparisons when a significant group × week interaction was detected. Bone degeneration, arthritis severity, duration of inflammation, and leg closure rate were analyzed using unpaired Student’s t-tests. VOR data were analyzed using a two-way mixed-effects model, the MRI-derived endolymphatic space area ratio using the Mann–Whitney U test, and serum type II collagen-specific autoantibody levels using one-way analysis of variance followed by Tukey’s multiple-comparison test.

Regarding Figure 6, the Normal, Con, Si, ConL, and SiL groups consisted of independent animals. Therefore, the description of a paired t-test in the previous version was incorrect and represented a description error in the Methods section. The data were analysed using one-way analysis of variance followed by Tukey’s multiple-comparison test. We have corrected this error in the revised manuscript.

These revisions clarify the statistical methods used for each dataset. We also revised the corresponding “Results” sections (lines 229-235, 247-249 on page 11, lines 271-277 on pages 12-13, lines 314-317, 321-328 on pages 14-15) and Figure legends (Fig.1a, 1f, 3b and 6) to reflect the revised statistical analyses and their results.

4. The main conclusion of this study is based on negative results (no significant differences in VOR, histology, or MRI). However, no information is provided regarding sample size justification or statistical power. Therefore, it is difficult to determine whether there is truly no treatment effect or whether the study is underpowered (Type II error). Since the main conclusion relies on negative findings, this point should be discussed. In addition, the present data indicate that no significant difference was detected; they do not prove that the treatment has no effect. The interpretation of the negative findings should therefore be more cautious throughout the Discussion.

Response 4

Thank you for this important comment. We agree that the absence of statistically significant differences does not establish the absence of a treatment effect and that insufficient statistical power should be considered when interpreting negative findings. A formal a priori power analysis was not performed in the present study. We have therefore added this point as a limitation in the “Discussion” section (lines 399-413 on pages 17-18) and revised the interpretation of the negative findings throughout the manuscript.

Specifically, we now clarify that the absence of statistically significant differences indicates that no detectable differences were observed under the conditions of the present study, rather than demonstrating that the Si-based agent has no effect. We have also revised the wording in the Discussion to avoid overinterpretation of the VOR, MRI, and histological findings.

5. Arthritis scoring is subjective and susceptible to observer bias. The manuscript does not describe whether the assessment was performed in a blinded manner or how many evaluators were involved. Please provide these methodological details.

Response 5

Thank you for this important comment. The type II collagen-induced arthritis model was established by Japan SLC, using a well-established and widely used protocol. Arthritis scoring was performed by an evaluator who was independent of the personnel responsible for the treatment and was unaware of the group assignments and individual treatment information. We have added these methodological details to the “Animals and model preparation” subsection of the “Materials and Methods” section (lines 105-106, 120-123 on pages 5-6).

6. MRI was used to evaluate endolymphatic hydrops. However, the previous study did not demonstrate obvious hydrops in the CIA model. Since no normal control group was included in the present study, the rationale for including MRI as a major outcome measure should be explained more clearly in the Introduction or Discussion.

Response 6

Thank you for this important comment. As noted by the reviewer, our previous study [Ref No. 9 in the text] did not demonstrate obvious endolymphatic hydrops when CIA mice were compared with WT mice. Therefore, we agree that the rationale for evaluating MRI as a measure of endolymphatic hydrops in the present study was limited.

In the present study, our purpose was to examine whether long-term administration of the Si-based agent altered the endolymphatic space in CIA mice, rather than to specifically evaluate endolymphatic hydrops. We have therefore revised the description of the MRI assessment to focus on changes in the endolymphatic space and clarified this rationale in the “Discussion” section (lines 377-381, 387-389, 399 on page 17). We also revised the interpretation of the MRI methods and findings accordingly (lines 191-197 on page 9, lines 294, 297-298 on page 13, 304-307 on page 14).

Minor comments

1. Figure 6 presents ELISA data for serum type II collagen antibody levels, but no ELISA method is described in the Methods section. Please add the experimental details, including the assay kit, procedures, and analysis. In addition, normal mice are included in Figure 6, but the Methods section does not describe these animals. Please clarify how the normal controls were prepared and analyzed.

Response 1

Thank you for this important comment. We have added the detailed ELISA procedure for measuring serum type II collagen antibody levels to the “Materials and Methods” section (lines 199-207 on pages 9-10), including the assay kit, experimental procedure, and data analysis. We have also clarified the preparation and analysis of the normal control mice shown in Figure 6 and added the relevant information to the “Animals and model preparation” subsection of “Materials and Methods” section (lines 102-104 on page 6).

2. The description of the VOR experiment in the Methods section is largely identical to that in the previous publication. I suggest citing the previous paper and describing only the information necessary for the present study.

Response 2

Thank you for this helpful comment. We have revised the VOR experiment section of the “Materials and Methods” section (lines 132-156 on pages 7-8) to avoid unnecessary duplication with our previous publication. We now cite the previous paper [Ref No. 9 in the text] and provide only the methodological details necessary for the present study.

3. The section entitled “Eye movement recording” (around Line 171) appears to describe the static tilt experiment. However, no static tilt results are presented in this manuscript. If this section is not relevant, it should be removed.

Response 3

Thank you for pointing this out. We completely overlooked the inclusion of this section during manuscript preparation. We agree that the “Eye movement recording” subsection describes the static tilt experiment, which was not performed or reported in the present study. We have therefore removed this section from the “Materials and Methods” section.

4. The Abstract is unbalanced. Most of the Abstract is devoted to the Background, whereas the Methods and Results are too brief. The study design, evaluation methods, and main findings should be described more clearly. A structured Abstract (or a similar format) would improve readability. The major negative findings should also be clearly stated.

Response 4

Thank you for this helpful comment. We have substantially revised the “Abstract” section to improve its balance and readability. The Background has been shortened, while the study design, evaluation methods, and main findings have been described more clearly. We have also explicitly stated the major negative findings regarding vestibular function-related outcomes.

Overall, this study addresses an interesting topic, and the finding that the Si-based agent improved arthritis in the CIA model is valuable. However, several important limitations in the study design, statistical analysis, and interpretation of the negative findings should be addressed before the conclusions can be fully supported. In particular, the conclusions regarding vestibular dysfunction should be limited to what is supported by the present data. Revision of the Methods and Discussion would substantially improve the manuscript.

Response

Thank you for the valuable and constructive comments. We have carefully addressed the concerns regarding the study design, statistical analysis, and interpretation of the findings, and revised the Methods and Discussion accordingly. We have also appropriately limited our conclusions regarding vestibular dysfunction to what is supported by the present data.

Attachment Submitted filename: Response_to_ Reviewers .docx

# Peer review: Decision Letter 1

**DOI:** 10.1371/journal.pone.0361954.r003

**Authors:** Toru Miwa

12 Oct 2026

Dear Dr. Koyama,

Thank you for submitting your manuscript to PLOS One. After careful consideration, we feel that it has merit but does not fully meet PLOS One’s publication criteria as it currently stands. Therefore, we invite you to submit a revised version of the manuscript that addresses the points raised during the review process.

Please submit your revised manuscript by Nov 11 2026 11:59PM. If you will need more time than this to complete your revisions, please reply to this message or contact the journal office at plosone@plos.org. When you're ready to submit your revision, log on to https://www.editorialmanager.com/pone/ and select the 'Submissions Needing Revision' folder to locate your manuscript file.

- A letter that responds to each point raised by the academic editor and reviewer(s). You should upload this letter as a separate file labeled 'Response to Reviewers'.
- A marked-up copy of your manuscript that highlights changes made to the original version. You should upload this as a separate file labeled 'Revised Manuscript with Track Changes'.
- An unmarked version of your revised paper without tracked changes. You should upload this as a separate file labeled 'Manuscript'.

If you would like to make changes to your financial disclosure, please include your updated statement in your cover letter. Guidelines for resubmitting your figure files are available below the reviewer comments at the end of this letter.

If applicable, we recommend that you deposit your laboratory protocols in protocols.io to enhance the reproducibility of your results. Protocols.io assigns your protocol its own identifier (DOI) so that it can be cited independently in the future. For instructions see: https://journals.plos.org/plosone/s/submission-guidelines#loc-laboratory-protocols. Additionally, PLOS One offers an option for publishing peer-reviewed Lab Protocol articles, which describe protocols hosted on protocols.io. Read more information on sharing protocols at https://plos.org/protocols?utm_medium=editorial-email&utm_source=authorletters&utm_campaign=protocols.

As the corresponding author, your ORCID iD is verified in the submission system and will appear in the published article. PLOS supports the use of ORCID, and we encourage all coauthors to register for an ORCID iD and use it as well. Please encourage your coauthors to verify their ORCID iD within the submission system before final acceptance, as unverified ORCID iDs will not appear in the published article. *Only*  the individual author can complete the verification step; PLOS staff *cannot*  verify ORCID iDs on behalf of authors.

We look forward to receiving your revised manuscript.

Kind regards,

Toru Miwa

Academic Editor

PLOS One

**Journal Requirements:**

1. If the reviewer comments include a recommendation to cite specific previously published works, please review and evaluate these publications to determine whether they are relevant and should be cited. There is no requirement to cite these works unless the editor has indicated otherwise.

2. Please review your reference list to ensure that it is complete and correct. If you have cited papers that have been retracted, please include the rationale for doing so in the manuscript text, or remove these references and replace them with relevant current references. Any changes to the reference list should be mentioned in the rebuttal letter that accompanies your revised manuscript. If you need to cite a retracted article, indicate the article’s retracted status in the References list and also include a citation and full reference for the retraction notice.

[Note: HTML markup is below. Please do not edit.]

Reviewers' comments:

Reviewer's Responses to Questions

**Comments to the Author**

Reviewer #1: (No Response)

**********

2. Is the manuscript technically sound, and do the data support the conclusions?

Reviewer #1: Partly

**********

3. Has the statistical analysis been performed appropriately and rigorously?

Reviewer #1: No

**********

4. Have the authors made all data underlying the findings in their manuscript fully available?

The PLOS Data policy

Reviewer #1: Yes

**********

5. Is the manuscript presented in an intelligible fashion and written in standard English?

Reviewer #1: Yes

**********

Reviewer #1: The authors have adequately addressed most of my previous comments, and the manuscript has improved substantially. However, a few issues remain.

Major comments

1. Multiple comparisons (Fig. 1a)

The use of a linear mixed-effects model is an improvement. However, the subsequent Student's t-tests at individual time points were performed without adjustment for multiple comparisons. I suggest omitting these post hoc comparisons and focusing on the significant group × week interaction. Alternatively, an appropriate multiple-comparison adjustment should be applied.

2. Statistical analysis of arthritis-related outcomes (Figs. 1 and 2)

The Methods section states that unpaired Student's t-tests were used for bone degeneration, arthritis severity, duration of inflammation, and leg closure rate, but the corresponding p-values are not clearly reported. Since bone degeneration, arthritis severity, and leg closure rate are binary outcomes, Fisher's exact test would be more appropriate, particularly given the small sample size (n = 9 per group). Please revise the analyses accordingly and report the results. Even if some comparisons are not statistically significant, the observed differences between groups should be described and interpreted cautiously.

3. Interpretation of the findings

Although the Discussion has been revised appropriately, some statements in the Results remain overly definitive. For example, the heading at lines 279–280 and the concluding statement at lines 291–293 imply that the Si-based agent was ineffective. However, the absence of detectable differences does not establish the absence of a treatment effect. Furthermore, the qualitative histological findings do not directly support conclusions about vestibular function. Please revise these statements and ensure consistent interpretation throughout the manuscript.

Minor comments

1. Line 248: “Fig. 1g” should be corrected to “Fig. 1f”.

2. Lines 569–572: The descriptions of Figures 1b and 1c appear to be reversed. Please correct the figure legends.

**********

what does this mean?). If published, this will include your full peer review and any attached files.

If you choose “no”, your identity will remain anonymous but your review may still be made public.

**Do you want your identity to be public for this peer review?**  For information about this choice, including consent withdrawal, please see our Privacy Policy

Reviewer #1: No

**********

[NOTE: If reviewer comments were submitted as an attachment file, they will be attached to this email and accessible via the submission site. Please log into your account, locate the manuscript record, and check for the action link "View Attachments". If this link does not appear, there are no attachment files.]

To ensure your figures meet our technical requirements, please review our figure guidelines: https://journals.plos.org/plosone/s/figures

You may also use PLOS’s free figure tool, NAAS, to help you prepare publication quality figures: https://journals.plos.org/plosone/s/figures#loc-tools-for-figure-preparation.

NAAS will assess whether your figures meet our technical requirements by comparing each figure against our figure specifications.

# Author comment: Author response to Decision Letter 2

**DOI:** 10.1371/journal.pone.0361954.r004

18 Oct 2026

Editor’s comment

Thank you for submitting your manuscript to PLOS One. After careful consideration, we feel that it has merit but does not fully meet PLOS One’s publication criteria as it currently stands. Therefore, we invite you to submit a revised version of the manuscript that addresses the points raised during the review process.

Response

Thank you for the opportunity to revise our manuscript. We have carefully addressed the reviewers’ comments and revised the manuscript accordingly.

Reviewer #1: The authors have adequately addressed most of my previous comments, and the manuscript has improved substantially. However, a few issues remain.

Response

Thank you for your positive assessment of our revised manuscript. We have carefully addressed the remaining comments and revised the manuscript accordingly.

Major comments

1. Multiple comparisons (Fig. 1a)

The use of a linear mixed-effects model is an improvement. However, the subsequent Student's t-tests at individual time points were performed without adjustment for multiple comparisons. I suggest omitting these post hoc comparisons and focusing on the significant group × week interaction. Alternatively, an appropriate multiple-comparison adjustment should be applied.

Response 1

Thank you for this important comment. The between-group comparisons at individual time points were performed based on the significant group × week interaction, with adjustment for multiple comparisons. We have clarified the statistical procedure in both the “Results” section (lines 239-240 on page 14) and the corresponding Fig1a legend to ensure consistency in the presentation of the findings.

2. Statistical analysis of arthritis-related outcomes (Figs. 1 and 2)

The Methods section states that unpaired Student's t-tests were used for bone degeneration, arthritis severity, duration of inflammation, and leg closure rate, but the corresponding p-values are not clearly reported. Since bone degeneration, arthritis severity, and leg closure rate are binary outcomes, Fisher's exact test would be more appropriate, particularly given the small sample size (n = 9 per group). Please revise the analyses accordingly and report the results. Even if some comparisons are not statistically significant, the observed differences between groups should be described and interpreted cautiously.

Response 2

Thank you for this valuable suggestion. We agree that Fisher’s exact test is more appropriate than the unpaired Student’s t-test for comparing binary outcomes, particularly given the small sample size. Accordingly, we reanalyzed bone degeneration, arthritis severity, and arthritis-related leg closure using Fisher’s exact test. The incidence of bone degeneration was significantly lower in the Si group than in the Con group (77.8% vs. 11.1%, p = 0.0152). In contrast, the differences in the proportion of mice with severe arthritis (77.8% vs. 55.6%, p = 0.6199) and arthritis-related leg closure (77.8% vs. 22.2%, p = 0.0567) were not statistically significant. The duration of severe arthritis, defined as the number of weeks during which the arthritis index was ≥4, was compared using the Wilcoxon rank-sum test. Although this duration was numerically shorter in the Si group, the difference was not statistically significant (p = 0.0753).

We have revised the “Statistical Analysis” subsection of the “Materials & Methods” (lines 212, 216-220 on page 13) and “Results” (lines 243-254 on page 15, 261-262, lines 270-273 on page 16), “Discussion” section (lines 341-345 on page 20), and Fig.1,2 legends to reflect the updated statistical analyses and results. We have also revised the interpretation to distinguish statistically significant findings from numerical differences that did not reach statistical significance.

3. Interpretation of the findings

Although the Discussion has been revised appropriately, some statements in the Results remain overly definitive. For example, the heading at lines 279–280 and the concluding statement at lines 291–293 imply that the Si-based agent was ineffective. However, the absence of detectable differences does not establish the absence of a treatment effect. Furthermore, the qualitative histological findings do not directly support conclusions about vestibular function. Please revise these statements and ensure consistent interpretation throughout the manuscript.

Response 3

Thank you for this valuable comment. We have revised the Results section to avoid overly definitive statements regarding the effects of the Si-based agent. Specifically, we have revised the relevant headings and concluding statements to clarify that the absence of statistically significant differences does not establish the absence of a treatment effect. We have also revised the description of the histological findings to limit the conclusions to the observed structural changes, without inferring effects on vestibular function. These revisions have been made consistently throughout the “Results” section (line 276 on page 16, lines 290-293 on page 17, lines 301-302, 307-309, 316-317 on page 18).

Minor comments

1. Line 248: “Fig. 1g” should be corrected to “Fig. 1f”.

Response 1

Thank you for pointing this out. We have corrected “Fig. 1g” to “Fig. 1f” in Line 257 of the revised manuscript.

2. Lines 569–572: The descriptions of Figures 1b and 1c appear to be reversed. Please correct the figure legends.

Response 2

Thank you for pointing this out. We have revised the figure legends to clarify that Fig. 1b shows the proportion of mice with an arthritis index of 4 or higher (arthritis severity), whereas Fig. 1c shows the proportion of mice with bone degeneration (arthritis index = 5). Because the Results section describes arthritis severity followed by bone degeneration, we have also switched the descriptions of Fig. 1b and Fig. 1c in the Figure legends to maintain consistency with the order of presentation in the “Results” section. The Figure legends have been corrected accordingly.

Attachment Submitted filename: Response to Reviewrers.docx

# Peer review: Decision Letter 2

**DOI:** 10.1371/journal.pone.0361954.r005

**Authors:** Toru Miwa

20 Oct 2026

Evaluation of the Efficacy of Si-based Agent in Rheumatoid Arthritis with Complicating vestibular impairment

PONE-D-26-31345R2

Dear Dr. Koyama,

We’re pleased to inform you that your manuscript has been judged scientifically suitable for publication and will be formally accepted for publication once it meets all outstanding technical requirements.

Within one week, you’ll receive an e-mail detailing the required amendments. When these have been addressed, you’ll receive a formal acceptance letter and your manuscript will be scheduled for publication.

An invoice will be generated when your article is formally accepted. Please note, if your institution has a publishing partnership with PLOS and your article meets the relevant criteria, all or part of your publication costs will be covered. Please make sure your user information is up-to-date by logging into Editorial Manager at Editorial Manager® and clicking the ‘Update My Information' link at the top of the page. For questions related to billing, please contact billing support.

If your institution or institutions have a press office, please notify them about your upcoming paper to help maximize its impact. If they’ll be preparing press materials, please inform our press team as soon as possible -- no later than 48 hours after receiving the formal acceptance. Your manuscript will remain under strict press embargo until 2 pm Eastern Time on the date of publication. For more information, please contact onepress@plos.org.

Kind regards,

Toru Miwa

Academic Editor

PLOS One

Additional Editor Comments (optional):

Reviewers' comments:

Reviewer's Responses to Questions

**Comments to the Author**

Reviewer #1: (No Response)

**********

2. Is the manuscript technically sound, and do the data support the conclusions?

Reviewer #1: (No Response)

**********

3. Has the statistical analysis been performed appropriately and rigorously?

Reviewer #1: (No Response)

**********

4. Have the authors made all data underlying the findings in their manuscript fully available?

The PLOS Data policy

Reviewer #1: (No Response)

**********

5. Is the manuscript presented in an intelligible fashion and written in standard English?

Reviewer #1: (No Response)

**********

Reviewer #1: The authors have adequately addressed my previous concerns, and the manuscript has improved substantially.

I have only one minor comment regarding the statistical analysis. Although the authors state that multiple-comparison adjustment was applied to the post hoc comparisons in Fig. 1a, the specific adjustment method is not described. Please specify the method used in the Statistical Analysis section to ensure reproducibility.

Otherwise, I have no further concerns and consider the manuscript suitable for publication.

**********

what does this mean?). If published, this will include your full peer review and any attached files.

If you choose “no”, your identity will remain anonymous but your review may still be made public.

**Do you want your identity to be public for this peer review?**  For information about this choice, including consent withdrawal, please see our Privacy Policy

Reviewer #1: No

**********

# Editor report: Acceptance letter

**DOI:** 10.1371/journal.pone.0361954.r006

**Authors:** Toru Miwa

PONE-D-26-31345R2

PLOS One

Dear Dr. Koyama,

I'm pleased to inform you that your manuscript has been deemed suitable for publication in PLOS One. Congratulations! Your manuscript is now being handed over to our production team.

At this stage, our production department will prepare your paper for publication. This includes ensuring the following:

* All references, tables, and figures are properly cited

* All relevant supporting information is included in the manuscript submission,

* There are no issues that prevent the paper from being properly typeset

You will receive further instructions from the production team, including instructions on how to review your proof when it is ready. Please keep in mind that we are working through a large volume of accepted articles, so please give us a few days to review your paper and let you know the next and final steps.

Lastly, if your institution or institutions have a press office, please let them know about your upcoming paper now to help maximize its impact. If they'll be preparing press materials, please inform our press team within the next 48 hours. Your manuscript will remain under strict press embargo until 2 pm Eastern Time on the date of publication. For more information, please contact onepress@plos.org.

You will receive an invoice from PLOS for your publication fee after your manuscript has reached the completed accept phase. If you receive an email requesting payment before acceptance or for any other service, this may be a phishing scheme. Learn how to identify phishing emails and protect your accounts at https://explore.plos.org/phishing.

If we can help with anything else, please email us at customercare@plos.org.

Thank you for submitting your work to PLOS One and supporting open access.

Kind regards,

PLOS One Editorial Office Staff

on behalf of

Dr. Toru Miwa

Academic Editor

PLOS One
